# Respiratory Health Status of Workers in a Bottling Factory in Benin City, Nigeria

**DOI:** 10.3390/ijerph15091919

**Published:** 2018-09-04

**Authors:** Sunday Omokiniovo Oghuvwu, Eruke E. Egbagbe, Joshua Oisezenome Aigbirior, Bright Ejakpovi Oniovokukor, Gregory E. Erhabor

**Affiliations:** 1 Department of Internal Medicine, University of Benin Teaching Hospital, Benin City 300271, Nigeria; eegbagbe@yahoo.com (E.E.E.); joaigbirior@yahoo.com (J.O.A.); sirniovo@gmail.com (B.E.O.); 2 Department of Internal Medicine, Obafemi Awolowo University Teaching Hospital, Ile Ife 220212, Nigeria; gregerhabor7@yahoo.com

**Keywords:** respiratory health, bottling factory workers, Nigeria

## Abstract

*Introduction*: There is a paucity of data on the respiratory health status of workers in bottling factories in Benin City, Nigeria. Such data will help to drive future studies and influence policy development on occupational health and safety in the country. This study assesses the respiratory symptoms and spirometric indices of exposed workers and controls. *Methods*: Respiratory symptoms and spirometric parameters of 18 workers on routine mandatory annual lung screening were assessed using the modified MRC (Medical Research Council) questionnaire and spirometer respectively, according to the European Respiratory Society and American Thoracic Society (ERS/ATS) guidelines. *Results*: The mean age of workers was 35.1 ± 6.7 years. Workers and controls were similar in age, sex, BMI (Body Mass Index) and health status (*p* > 0.05). Respiratory symptoms were significantly higher among workers compared to controls. Overall, the result was statistically significant in the variables of wheeze in a smoky or dusty environment, presence of at least one respiratory symptom, better symptoms at weekends and better symptoms during holidays (*p* < 0.05). In particular, 6 (33.3%) exposed workers had wheeze in a smoky or dusty environment, 9 (50.0%) exposed workers reported at least one respiratory symptom compared with 2 (11.1%) controls, 5 (27.8%) had better symptoms at weekends, and 7 (38.9%) had better symptoms at holidays (*p* < 0.05). Generally, the reported frequency of respiratory symptoms among exposed workers were: cough (22.2%), sputum production (5.6%), breathlessness (11.1%) and wheeze (44.4%). Similarly, workers had significantly lower spirometric indices than controls, particularly in forced expiratory volume in 1 s (FEV_1_), FEV_1_/forced vital capacity (FVC) ratio and forced expiratory flow between 25% and 75% of FVC (FEF 25–75%) measurements. *Conclusions*: This study provides evidence of adverse respiratory health effects among bottling factory workers which requires further investigation.

## 1. Introduction

The International Labour Organization (ILO) estimates that work-related diseases and occupational accidents account for 2 million preventable deaths worldwide. They estimate that there are approximately 270 million occupational accidents and 160 million occupational diseases each year, which equates to a lost global Gross Domestic Product (GDP) of four percent [1]. Workplace safety is thus an important aspect of occupational health that is attracting sustained public health campaign, particularly in many developing countries including Nigeria [2]. Following a situation analysis of the national Occupational Safety and Health (OSH) Systems in 2010, the ILO embarked on the implementation of a special project with the overall objective of reducing occupational accidents and work related morbidities in the country. However, nearly a decade down the line, under reporting of occupational accidents and diseases still persists [3]. One study attributed this to inadequate stakeholder investment in occupational health and safety programmes and lack of a national OSH code of practice in the country [4].

Furthermore, the ILO has stipulated standards for occupational safety towards the protection of workers [5]. However, there are indications of non-compliance in many developing countries, despite the abundance of evidence of significant economic and health benefits to both employers and employees [6,7]. In some cases, company-sponsored data are projected at the expense of credible scientific evidence [8,9]. The implication of this is unbridled exposure of workers to work-related morbidity and mortality. Thus, in the midst of competing social and political challenges, occupational health may be at risk of being relegated to the background if workers do not rise up to claim their rights [10]. The goal would be to maintain the delicate balance between work, health and productivity which is the cardinal thrust of principle 1 of the Rio Declaration on Environment and Development [11].

Work related hazards in diverse occupations have been well studied in developing countries. The majority of these studies centered on the agricultural, textile and workers in extractive industries, others concentrated on the transportation, tobacco, food and beverage industries [12,13,14,15,16,17,18]. Similarly, existing evidence show that occupational health hazards also feature among healthcare workers [19,20]. Further analysis of the methodology showed that no specific description of type of hazards was made and it is not clear whether the biohazards suffered included respiratory diseases. There is also evidence that bottling factory workers suffer hazard exposure in their workplace. However, the results differ in the hazard measured. For instance, Ologe et al. demonstrated a high level of noise-induced hearing loss among bottling factory workers in Ibadan [21]. In another study, Aliyu et al. found biohazard exposure rate of nearly 50% among workers in a bottling company [22]. However, data analysis show that respiratory health was not assessed and no objective tool of measurement of ill-health was used in the study. Clearly, limited attention has been paid to respiratory health of workers in bottling companies. There is therefore an urgent need for data on the respiratory health of bottling factory workers in Benin City, Nigeria. Such data will be leveraged on by policy makers to drive change and promote both a safe work environment and healthy workers in the area.

In this cross-sectional pilot study in Benin City, Midwestern Nigeria, we assessed the respiratory health of workers in a bottling factory in terms of respiratory symptoms and spirometric abnormalities.

## 2. Materials and Methods

### 2.1. Study Design and Setting

This was a descriptive cross-sectional pilot survey conducted at a specialized diagnostic center in Benin City, Southern Nigeria between April and May 2015. Benin City is the metropolitan capital of Edo State and about 322 km south of Lagos and 700 km south of Abuja, the Federal Capital City of Nigeria. The population of Benin City is 1,495,800, which is projected to be 1,682,158 in 2018 [23]. The diagnostic centre has state of the art facilities such as Spirometer, Electrocardiography, Echocardiography, X-ray machines, Mammography, Computer tomography, Doppler and ultrasound scans. The spirometer is operated by an ERS trained spirometer expert. The study is approved by The University of Benin Teaching Hospital (UBTH) ethics committee (protocol number: ADM/E 22/A/VOL. VII/1285).

### 2.2. Study Population

Workers in a bottling factory located in Benin City were recruited for the study. The factory has two main departments, namely production and administration. Notably, workers are employed directly into the production or administration sections and remain so till dismissal, resignation or retirement. Thus, duration of employment is equivalent to the duration of exposure. Workers in the production department were categorized into exposed or non-exposed based on their contact with caustic and syrup concentrate fumes. In the end, 18 workers fell into the exposed group. In compliance with company policy, these workers were sent for mandatory annual lung function tests and evaluation. We thus conducted a total population sampling of exposed workers (18). The control group were healthy male security personnel (*n* = 20) of the University of Benin teaching hospital (UBTH), which is about 500 m from the factory. This is to ensure a distinct population of workers (exposed workers versus controls) that differ only in exposure to factory fumes, in order to detect the effect of an exposure if present. Controls were similar to cases with regards to age, sex, BMI (Body Mass Index), nationality, and similar work hours (shift work).

### 2.3. Data Collection

A one-time assessment of respiratory symptoms and spirometry of workers was conducted at presentation. Respiratory symptoms of subjects and controls was assessed using a modified British Medical Research Council respiratory questionnaire [24]. Questionnaires were administered in English, as all the workers have at least secondary level education and basic understanding of oral and written English. Questionnaire assessed recall of respiratory symptoms in the last 1 to 3 years. Spirometric indices were measured using a portable Spirometer (Spirovit SP-1, SCHILLER-AG, AH gasse 68, post fach, 6340 Barr, Switzerland) according to ERS/ATS guidelines [25]. Measurements were performed with subjects in sitting position with a nose clip to prevent air leakage through the nostrils. Forced expiratory maneuvers were repeated until three accepted and reproducible readings were recorded. The maximum of the three was determined, and values compared with predicted values for age, sex, height and ethnic group. Daily calibration of the Spirometer with a 3.00 L syringe was done according to the manufacturer’s recommendations. Each spirogram was carefully scrutinized by the lead author according to ERS/ATS guidelines, for acceptability and reproducibility. Predicted values were calculated by the reference equations from NHANES (National Health and Nutrition Examination Survey) III with a 12% reduction for African ethnic group [26].

With the aid of a spirometer, the forced expiratory volume in 1 s (FEV_1_), forced vital capacity (FVC), FEV_1_/FVC ratio, peak expiratory flow (PEF) and forced expiratory flow between 25% and 75% of FVC (FEF 25–75%) was determined for each subject and control. FEV_1_ and FVC < 80% predicted indicate restrictive lung disease while FEV_1_/FVC ratio < 70% predicted connotes an obstructive pattern. Mixed or combined (i.e., restrictive and obstructive) disease is demonstrated when both parameters are decreased in the same subject (FVC < 80%; FEV_1_/FVC < 70%) [27]. All the ventillatory function tests were carried out between the hours of 900 h to 1200 h Weight was measured with light clothing, without footwear, using Avery scales (Avery Berkel, West Midlands, UK, 2003). Standing height was measured without footwear, with feet together and the patient standing tall with eyes level and looking straight, using a wall mounted stadiometer. Blood Pressure was measured with a mercury sphygmomanometer according to standard procedure.

### 2.4. Data Analysis

Statistical analysis was performed with IBM SPSS version 22 (SPSS Inc., Chicago, IL, USA). Continuous variables were expressed in mean and standard deviation and categorical variables in proportions. Differences in means between exposed workers and controls will be compared with Independent *t* test for continuous variables. Fisher’s exact test was used for categorical variables because of the very low cell count. Chi square was used to test for association. Statistical significance was set at 5%.

## 3. Results

### 3.1. Sociodemographics Characteristics

Of the 18 exposed workers who presented for their annual lung screening, 16 (88.8%) completed spirometry, 1 (5.6%) declined the procedure, another 1 (5.6%) had an absolute contraindication (micrognathia) to spirometry. Two of the spirograms did not meet acceptability criteria, leaving a net number of 14 (77.7%) spirograms for analysis. As shown in Table 1, the mean age of the bottling factory workers was 35.1 ± 6.7 years compared to 36.1 ± 7.3 years among controls. There were no current smokers among the cases or the controls. The median duration of employment amongst bottling factory workers was 11.0 years with a range of 4–28 years. The mean systolic and diastolic blood pressures of exposed workers were 124.12 mmHg and 84.12 mmHg respectively. Eight (44.44%) workers had systolic or diastolic blood pressure of at least 140 mmHg and 90 mmHg respectively, suggesting a high frequency of systemic hypertension. However, there was no statistically significant difference between workers and controls (see Table 1). 

### 3.2. Respiratory Symptoms

Table 2 depicts the frequency of respiratory symptoms among exposed workers and controls. Clearly, the frequency of respiratory symptoms was higher among exposed workers than controls. Overall, the result was statistically significant in the variables of wheeze in a smoky or dusty environment, presence of at least one respiratory symptom, better symptoms at weekends and better symptoms during holidays (*p* < 0.05). In particular, 6 (33.3%) exposed workers had wheeze in a smoky or dusty environment, 9 (50.0%) exposed workers reported at least one respiratory symptom compared with 2 (11.1%) of controls, 5 (27.8%) had better symptoms at weekends, and 7 (38.9%) had better symptoms at holidays (*p* < 0.05). Generally, the reported frequency of respiratory symptoms among exposed workers were: cough (22.2%), sputum production (5.6%), breathlessness (11.1%) and wheeze (44.4%).

### 3.3. Spirometric Indices and Arthropometry

As shown in Table 3, exposed workers had a lower BMI compared to controls (21.82 ± 2.71 versus 23.77 ± 3.61). The difference was however not statistically significant (*p* > 0.05). Similarly, spirometric indices were lower in exposed workers than in controls. However, only the difference in FEV_1_, FEV_1_/FVC ratio and FEF 25–75% reached statistical significance. No association was found between respiratory symptoms or spirometric indices with age, BMI, duration of employment or smoking status.

## 4. Discussion

This was a pilot study that assessed the respiratory health status of bottling factory workers. The data showed that workers and controls were comparable in age (35.1 vs. 36.1 years), sex and BMI (21.8 vs. 23.8), and the observation of non-statistical significance suggests that workers and controls were similar with regards to these variables. However, the study design and small sample size did not allow for matching, which could impact on the generalizability and accuracy of this study. Matching helps deal with sampling bias and control cofounders, thus enhancing the accuracy and precision of a study [28]. Average duration of employment of workers was 10 years, with a range of 4–28 years, which suggest that the workers had worked long enough for sufficient exposure. Meo et al. had demonstrated a direct proportional relationship between duration of exposure and development of chronic respiratory symptom [29]. The lack of association between duration of employment and abnormalities in pulmonary parameters found in this study may be due to the small sample size or the healthy worker effect, which may mean that very sick workers were not available for assessment and measurements of respiratory health status. In an earlier study Le Moual et al. have shown the two-way relationship between asthma and workplace, and recommended cautious interpretation of workplace adverse health events [30]. Evidence exist also that the prevalence of respiratory symptom in the general population increases with age [31]. However, this study observed that the majority of workers, though similar in age to controls, had higher prevalence of respiratory symptoms. This may imply that the cause of the respiratory symptoms is due to exposure to irritant fumes from the factory. In contrast, several other studies found no association between frequency of respiratory symptoms with age and duration of employment [32,33,34]. The conspicuous absence of smokers in the study was not surprising as this may be a reflection of the low prevalence of tobacco smoking in the area [35].

The results of this study also showed that bottling workers had a significantly higher prevalence of respiratory symptoms compared to controls. This may be due to the irritant effect of inhaled caustic fumes on the workers, poor ventilation in the production area and the lack of effective personal protective equipment. Awopeju et al. have shown that fume inhalation caused significantly higher odds of respiratory symptoms among a subset of female street cooks in Nigeria [36]. Particulate Matter analysis of the fumes, however, revealed benzene was the major culprit. Dries et al. had demonstrated inhalation injury to be a major cause of morbidity and mortality in developing countries [37]. In particular, the evidence showed that the injuries were higher where there is no proper ventilation. Evidence also abounds that use of Personal Protective Equipment (PPEs) not only reduce workplace hazards and exposure but improves job satisfaction [38]. However, results of one systematic review was less than conclusive on the comparative effectiveness of PPEs among workers [39]. Sadly, the absence of effective protection for workers still feature prominently in developing countries [40]. In some cases, non-utilization of PPEs despite high level of awareness of occupational hazards has been documented [41]. Although there were no bottling factory studies to compare with, the result however compares with that of workers in sister industries which demonstrated prominence of respiratory symptoms among exposed workers. In one Nigerian study, Ijadunola et al. estimated a prevalence of 54% among food grain workers [42]. Similarly, the high frequency of cough and breathlessness found in this study compares with result of studies from food processing workers. For instance, in Tanzania, Sakwari et al. estimated a prevalence of 25% for cough and breathlessness in coffee processing workers [43]. Similar results were also demonstrated among poultry food processing workers [44]. These results however differ from that of Ige et al. who demonstrated that sneezing and running nose was twice as common as cough and breathlessness (53.3% vs. 22.4%) in bakery factory workers in Nigeria [18]. These variations could be attributed to differences in the irritant exposed to and the inconsistencies in definition of cough and breathlessness across studies.

In addition, the observed lower spirometric indices among exposed workers compared to controls is consistent with results from sister food and beverage industries. In Egypt, Mohammadien et al. demonstrated similar reduced lung indices among bakery workers compared to controls [45]. They also noted the additive effect of smoking to this decline: an effect that was probably too little to cofound in our study. Similar results was demonstrated by van Rooy et al. among food flavouring workers in Europe [46]. Reduced spirometric indices among street cooks has also been shown in an earlier study in the area [36]. However, this comparison must be interpreted with caution, as only female respondents were assessed.

Finally, the authors included hypertension in this study to ascertain the similarity in comorbidity characteristics of participants. The results showed that hypertension was not uncommon among bottling factory workers. Eight (44.4%) workers had Systolic Blood Pressure (SBP) and Diastolic Blood Pressure (DBP) greater than or equal to 140 mmHg and 90 mmHg respectively which indicate a high frequency of hypertension among workers. Current guidelines have defined hypertension as SBP ≥ 140 mmHg and DBP ≥ 90 mmHg [47]. The lack of statistical significance between workers and controls suggest that workers and controls have a similar morbidity pattern. The results of this study however contrast with an earlier study by Ogunlesi et al. which noted a prevalence of 8% among factory workers in Ibadan, Nigeria [48]. This relatively lower prevalence could be attributed to the higher Blood Pressure cut-off (160/95 mmHg) that was used to define hypertension. Whether these findings are incidental or due to occupational exposures remains debatable, and evidence of association is urgently needed. Clearly, the clinical significance of the results of this study is the observation that exposed bottling factory workers are at risk of developing respiratory symptoms and spirometric abnormalities.

## 5. Conclusions

This is a pilot study in the area to evaluate respiratory health status of bottling factory workers. The result of the study suggest a high prevalence of respiratory symptoms and reduced spirometric indices among bottling factory workers. A large scale follow up study is needed to further characterize lung health among bottling factory workers.

### Strengths and Limitations

Both quantitative and qualitative variables were considered in assessing the respiratory health effects of caustic and concentrate fume inhalation among bottling factory workers. All the workers are from the same factory and share similar environmental characteristics. However, the study was limited by the lack of measurement of particulate matter (PM) and hence the inability to quantify exposure. Secondly, the small sample size and study design (cross-sectional/single-centre) will undermine causality and generalizability of the results of this study.

## Figures and Tables

**Table 1:** Socio-demographic characteristics.

Variable	Cases (*n* = 36)	Controls	*p*-Value
Age group			
25–29	3 (16.7)	2 (11.1)	0.955
30–34	8 (44.4)	8 (44.4)	
35–39	4 (22.2)	3 (16.7)	
40–44	1 (5.6)	1 (5.6)	
45–49	1 (5.6)	3 (16.7)	
50–54	1 (5.6)	1 (5.6)	
Mean ± SD age (years)	35.1 ± 6.7 years	36.1 ± 7.3 years	0.688
Sex			
Male	18 (100.0)	18 (100.0)	
Female	0 (0)	0 (0)	
Median (range) duration of exposure	11.0 (4.0–28) years	3.5 (0.5–12) years	0.001 *
Smoking status			
Yes	0 (0.0)	0 (0.0)	
No	18 (100.0)	18 (100.0)	
Mean ± SD Systolic BP	124.1 ± 18.5 mmHg	124. 7 ± 7.8 mmHg	0.907
Mean ± SD Diastolic BP	84.1 ± 10.3 mmHg	80.2 ± 4.9 mmHg	0.159

* Significant *p* < 0.05; SD = standard deviation; BP = Blood Pressure.

**Table 2:** Respiratory Symptoms among Bottling Factory Workers and Controls.

Symptoms	Bottling Factory Workers *n* (%)	Controls *n* (%)	*p*-Value
Cough during the day or night	3 (16.7)	1 (5.6)	0.603
Cough on most days as much as three months a year	1 (5.6)	0 (0.0)	1.000
Sputum production	1 (5.6)	1(5.6)	1.000
Difficulty with breathing	2 (11.1)	0 (0.0)	0.486
Early morning wheeze	2 (11.1)	0 (0.0)	0.486
Wheeze in a smoky or dusty place	6 (33.3)	0 (0.0)	0.019 *
Presence of at least one respiratory symptom	9 (50.0)	2 (11.1)	0.011 *
Better symptoms at weekends	5 (27.8)	0 (0.0)	0.045 *
Better symptoms during holidays	7 (38.9)	0 (0.0)	0.008 *

* Significant *p* < 0.05.

**Table 3:** Anthropometric and Spirometric Values of Bottling Factory Workers and Controls.

Variable	Bottling Factory Workers (*n* = 14) Mean ± SD	Controls (*n* = 18) Mean ± SD	*p*-Value
Height (m)	1.78 ± 0.06	1.74 ± 0.06	0.10
Weight (kg)	68.57 ± 7.52	71.61 ± 13.02	0.41
BMI (kg/m^2^	21.82 ± 2.71	23.77 ± 3.61	0.10
FVC (L)	3.69 ± 1.25	4.04 ± 0.63	0.31
FVC predicted (L)	4.60 ± 0.27	4.37 ± 0.40	0.06
FVC %	79.78 ± 26.69	92.20 ± 8.56	0.07
FEV_1_ (L)	2.81 ± 0.86	3.41 ± 0.57	0.03 *
FEV_1_ predicted (L)	3.82 ± 0.31	3.61 ± 0.33	0.07
FEV_1_ %	73.29 ± 22.07	94.11 ± 11.39	0.00
FEV_1_/FVC (%)	78.17 ± 8.50	84.31 ± 7.15	0.03 *
FEV_1_/FVC predicted (%)	82.91 ± 2.67	82.60 ± 2.12	0.72
FEV_1_/FVC %	94.32 ± 10.21	102.01 ± 7.29	0.02
FEF_25–75_ (L/s)	2.71 ± 0.74	3.62 ± 0.83	0.00 *
FEF_25–75_ predicted (L/s)	4.65 ± 0.34	4.52 ± 0.33	0.30
FEF_25–75_ %	58.25 ± 15.23	79.62 ± 15.85	0.00
PEFR (L/min)	452.57 ± 118.18	517.83 ± 97.92	0.10
PEFR predicted (L/min)	573.64 ± 29.79	556.19 ± 28.72	0.10
PEFR %	79.09 ± 21.12	92.69 ± 14.10	0.04

* Significant *p* < 0.05.

## References

[1] International Labour Organization Facts on Safe Work. www.ilo.org/safework.

[2] Hämäläinen P., Takala J., Saarela K.L. (2006). Global estimates of occupational accidents. Saf. Sci..

[3] International Labour Organization (2016). Nigeria Country Profile on Occupatinal Safety and Health.

[4] Akinwale A.A., Olusanya O.A. (2016). Implications of Occupational Health and Safety Intelligence in Nigeria. J. Glob. Healthc. Syst..

[5] Niu S. (2010). Ergonomics and occupational safety and health: An ILO perspective. Appl. Ergon..

[6] LaDou J. (2003). International occupational health. Int. J. Hyg. Environ. Health.

[7] Henke R.M., Carls G.S. (2010). Short, M.E. The relationship between health risks and health and productivity costs among employees at Pepsi Bottling Group. J. Occup. Environ. Med..

[8] Tóthová L., Hodosy J., Mettenburg K. (2013). No harmful effect of different Coca-cola beverages after 6 months of intake on rat testes. Food Chem. Toxicol..

[9] The Lancet (2009). Myths and morality at Coca-Cola. Lancet.

[10] Ngwama J.C. (2016). Framework for Occupational Health and Safety in Nigeria: The implication for the trade union movement. J. Econ. Sustain. Dev..

[11] WHO (2001). United Nations Conference on Environment and Development, Rio de Janeiro, 1992.

[12] Kwon S.C., Lee S.-J., Jeong M. (2012). Work-related hazards among farmers. J. Korean Med. Assoc..

[13] Zuskin E., Mataija M., Pokrajac D., Schachter E.N., Witek J. (1989). Respiratory function in animal food processing workers. Am. J. Ind. Med..

[14] Awodele O., Popoola T.D., Ogbudu B.S., Akinyede A., Coker H.A.B., Akintonwa A. (2014). Occupational hazards and safety measures amongst the paint factory workers in Lagos, Nigeria. Saf. Health Work.

[15] Mojiminiyi F.B.O., Merenu I.A., Ibrahim M.T.O., Njoku C.H. (2008). The effect of cement dust exposure on haematological and liver function parameters of cement factory workers in Sokoto, Nigeria. Niger. J. Physiol. Sci..

[16] Ekpenyong C.E., Ettebong E.O., Akpan E.E., Samson T.K., Daniel N.E. (2012). Urban city transportation mode and respiratory health effect of air pollution: A cross-sectional study among transit and non-transit workers in Nigeria. BMJ Open.

[17] Uitti J., Nordman H., Huuskonen M.S., Roto P., Husman K., Reiman M. (1998). Respiratory health of cigar factory workers. Occup. Environ. Med..

[18] Ige O.M., Awoyemi O.B. (2002). Respiratory symptoms and ventilatory function of the bakery workers in Ibadan, Nigeria. West Afr. J. Med..

[19] Bamidele J.O., Adeoye O.A., Ntaji M.I., Oladele E.A. (2014). Occupational hazards exposure and their resultant effects on hospital attendants in health care facilities of a Local Government Area in South-South, Nigeria. J. Environ. Occup. Sci..

[20] Ndejjo R., Musinguzu G., Yu X.J., Buregyeya E. (2015). Occupational Health Hazards among Healthcare workers in Kampala, Uganda. J. Environ. Public Health.

[21] Ologe F.E., Olajide T.G., Nwawolo C.C., Oyejola B.A. (2008). Deterioration of noise-induced hearing loss among bottling factory workers. J. Laryngol. Otol..

[22] Aliyu S.U., Auwal I.M. (2015). Occupational risk and Hazard Exposure, Knowledge of occupational health and safety practice and safety measures among workers of a Nigerian bottling company, Maiduguri, Bornu State. J. Harmonized Res. Med. Health Sci..

[23] National Bureau of Statistics (2017). Annual Abstract of Statistics, 2017.

[24] BMRC (1960). Standardized questionnaire on respiratory symptoms. BMJ Br. Med. J..

[25] Miller M.R., Hankinson J., Brusasco V. (2005). ATS/ERS Task Force: ATS/ERS Standardisation of spirometry. Eur. Respir. J..

[26] Hankinson J.L., Odencrantz J.R., Fedan K.B. (1999). Spirometric reference values from a sample of the general U.S. population. Am. J. Respir. Crit. Care Med..

[27] Pellegrino R., Viegi G., Brusasco V. (2005). Interpretative strategies for lung function tests. Eur. Respir. J..

[28] Hulley S.B., Cummings S.R., Browner W.S., Grady D.G., Newman T.B. (2007). Designing Clinical Research.

[29] Meo S.A., Al-Drees A.M., Al Masri A.A., Al Rouq F., Azeem M.A. (2013). Effect of duration of exposure to cement dust on respiratory function of non-smoking cement mill workers. Int. J. Environ. Res. Public Health.

[30] Le Moual N., Kauffmann F., Eisen E.A., Kennedy S. (2008). The healthy worker effect in asthma: Work may cause asthma, but asthma may also influence work. Am. J. Respir. Crit. Care Med..

[31] Eagan T.M.L., Bakke P.S., Eide G.E., Gulsvik A. (2002). Incidence of asthma and respiratory symptoms by sex, age and smoking in a community study. Eur. Respir. J..

[32] doPico G.A., Reddan W., Flaherty D., Tsiatis A., Peters M., Rao P., Rankin J. (1977). Respiratory abnormalities among grain handlers: A. clinical, physiologic, and immunologic study. Am. Rev. Respir. Dis..

[33] Chan-Yeung M., Schulzer M., Maclean L., Dorkhen E., Grzbowski S. (1980). Epidemiologic health survey of grain elevator workers in British Columbia. Am. Rev. Respir. Dis..

[34] Broder I., Hutcheon M.A., Mintz S., Davies G., Leznoff A., Thomas P., Corey P. (1984). Changes in respiratory variables of grain handlers and civil workers during their initial months of employment. Br. J. Ind. Med.

[35] Adeniji F., Bamgboye E., Van Walbeek C. (2016). Smoking in Nigeria:Estimates from the Global Adult Tobacco Survey (GATS) 2012. J. Sci. Res. Rep..

[36] Awopeju O.F., Nemery B., Afolabi O.T., Poels K., Vanoirbeek J., Obaseki D.O., Adewole O.O., Lawin H.A., Vollmer W., Erhabor G.E. Biomas smoke exposure as an occupational risk: Cross-sectional study of respiratory health of women working as street cooks in Nigeria. Occup. Environ. Med..

[37] Dries D.J., Endorf F.W. (2013). Inhalation injury: Epidemiology, pathology, treatment strategies. Scand. J. Trauma Resusc. Emerg. Med..

[38] Wagner H., Kim A.J., Gordon L. (2013). Relationship between Personal Protective Equipment, Self-Efficacy, and Job Satisfaction of Women in the Building Trades. J. Constr. Eng. Manag..

[39] Hersi M., Stevens A., Quach P., Hamel C., Thavorn K., Garritty C., Skidmore B., Vallenas C., Norris S.L., Egger M. (2015). Effectiveness of Personal Protective Equipment for Healthcare Workers Caring for Patients with Filovirus Disease: A Rapid Review. PLoS ONE.

[40] Izudi J., Ninsiima V., Alege J.B. (2017). Use of Personal Protective Equipment among Building Construction Workers in Kampala, Uganda. J. Environ. Public Health.

[41] Sabitu K., Illiyasu Z., Dauda M.M. (2009). Awareness of occupational hazards and utilization of safety measures among welders in Kaduna metropolis, Northern Nigeria. Ann. Afr. Med..

[42] Ijadunola K.T., Erhabor G.E., Onayade A.A., Ijadunola M.Y., Fatusi A.O., Asuzu M.C. (2004). Prevalence of respiratory symptoms among wheat flour mill workers in Ibadan, Nigeria. Am. J. Ind. Med..

[43] Sakwari G., Bråtveit M., Mamuya S.H., Moen B.E. (2011). Dust exposure and chronic respiratory symptoms among coffee curing workers in Kilimanjaro: A cross sectional study. BMC Pulm. Med..

[44] Zuskin E., Kanceljak B., Mustajbegovic J., Schachter E.N., Stilinovic L. (1994). Respiratory symptoms and immunological status in poultry food processing workers. Int. Arch. Occup. Environ. Health.

[45] Mohammadien H.A., Hussein M.T., El-Sokkary R.T. (2013). Effects of exposure to flour dust on respiratory symptoms and pulmonary function of mill workers. Egypt. J. Chest Dis. Tuberc..

[46] van Rooy F.G.B.G.J., Smit L.A.M., Houba R., Zaat V.A.C., Rooyackers J.M., Heederik D.J.J. (2009). A cross-sectional study of lung function and respiratory symptoms among chemical workers producing diacetyl for food flavourings. Occup. Environ. Med..

[47] Hypertension: The Silent Killer: Updated JNC-8 Guideline. https://cdn.ymaws.com/www.aparx.org/resource/resmgr/CEs/CE_Hypertension_The_Silent_K.pdf.

[48] Ogunlesi A., Osotimehin B., Abbiyessuku F. (1991). Blood pressure and educational level among factory workers in Ibadan, Nigeria. J. Hum. Hypertens..

